# The Saving and Empowering Young Lives in Europe (SEYLE) Randomized Controlled Trial (RCT): methodological issues and participant characteristics

**DOI:** 10.1186/1471-2458-13-479

**Published:** 2013-05-16

**Authors:** Vladimir Carli, Camilla Wasserman, Danuta Wasserman, Marco Sarchiapone, Alan Apter, Judit Balazs, Julio Bobes, Romuald Brunner, Paul Corcoran, Doina Cosman, Francis Guillemin, Christian Haring, Michael Kaess, Jean Pierre Kahn, Helen Keeley, Agnes Keresztény, Miriam Iosue, Ursa Mars, George Musa, Bogdan Nemes, Vita Postuvan, Stella Reiter-Theil, Pilar Saiz, Peeter Varnik, Airi Varnik, Christina W Hoven

**Affiliations:** 1 National Centre for Suicide Research and Prevention of Mental Ill-Health (NASP), Karolinska Institutet, Stockholm, Sweden; 2 WHO Collaborating Center for Research, Methods Development and Training in Suicide Prevention, Stockholm, Sweden; 3 Department of Child and Adolescent Psychiatry, Columbia University-New York State Psychiatric Institute, New York, USA; 4 Department of Health Sciences, University of Molise, Campobasso, Italy; 5 Feinberg Child Study Centre, Schneider Children’s Medical Centre, Tel Aviv University, Tel Aviv, Israel; 6 Vadaskert Child and Adolescent Psychiatric Hospital, Budapest, Hungary; 7 Institute of Psychology, Eötvös Loránd University, Budapest, Hungary; 8 Department of Psychiatry, University of Oviedo, Centro de Investigación Biomédica en Red de Salud Mental, CIBERSAM, Oviedo, Spain; 9 Section for Disorders of Personality Development, Clinic of Child and Adolescent Psychiatry, Heidelberg, Germany; 10 Centre of Psychosocial Medicine, University of Heidelberg, Heidelberg, Germany; 11 National Suicide Research Foundation, Cork, Ireland; 12 Clinical Psychology Department, Iuliu Hatieganu University of Medicine and Pharmacy, Cluj-Napoca, Romania; 13 Inserm CIC-EC, Nancy University Hospital, Nancy, France; 14 Research Division for Mental Health, University for Medical Information Technology (UMIT), Hall in Tirol, Austria; 15 Department of Psychiatry, Centre Hospitalo-Universitaire de Nancy, Université ed Lorraine, Nancy, France; 16 Semmelweis University, School of Ph.D. Studies, Budapest, Hungary; 17 Slovene Center for Suicide Research, UP IAM, University of Primorska, Koper, Slovenia; 18 Clinical Ethics Support & Accompanying Research, University Hospital Basel, Basel, Switzerland; 19 Psychiatric Clinics of the University Basel, IBMB, University of Basel, Basel, Switzerland; 20 Estonian-Swedish Mental Health & Suicidology Institute, Ctr. Behav. & Hlth. Sci, Tallinn University, Tallinn, Estonia; 21 Department of Epidemiology, Mailman School of Public Health, Columbia University, New York, USA

**Keywords:** SEYLE, Mental Health Promotion, Suicide prevention, Promotion, Well-being, Adolescents, Schools, RCT, Intervention, ProfScreen, QPR, Awareness

## Abstract

**Background:**

Mental health problems and risk behaviours among young people are of great public health concern. Consequently, within the VII Framework Programme, the European Commission funded the Saving and Empowering Young Lives in Europe (SEYLE) project. This Randomized Controlled Trial (RCT) was conducted in eleven European countries, with Sweden as the coordinating centre, and was designed to identify an effective way to promote mental health and reduce suicidality and risk taking behaviours among adolescents.

**Objective:**

To describe the methodological and field procedures in the SEYLE RCT among adolescents, as well as to present the main characteristics of the recruited sample.

**Methods:**

Analyses were conducted to determine: 1) representativeness of study sites compared to respective national data; 2) response rate of schools and pupils, drop-out rates from baseline to 3 and 12 month follow-up, 3) comparability of samples among the four Intervention Arms; 4) properties of the standard scales employed: Beck Depression Inventory, Second Edition (BDI-II), Zung Self-Rating Anxiety Scale (Z-SAS), Strengths and Difficulties Questionnaire (SDQ), World Health Organization Well-Being Scale (WHO-5).

**Results:**

Participants at baseline comprised 12,395 adolescents (M/F: 5,529/6,799; mean age=14.9±0.9) from Austria, Estonia, France, Germany, Hungary, Ireland, Israel, Italy, Romania, Slovenia and Spain. At the 3 and 12 months follow up, participation rates were 87.3% and 79.4%, respectively. Demographic characteristics of participating sites were found to be reasonably representative of their respective national population. Overall response rate of schools was 67.8%. All scales utilised in the study had good to very good internal reliability, as measured by Cronbach’s alpha (BDI-II: 0.864; Z-SAS: 0.805; SDQ: 0.740; WHO-5: 0.799).

**Conclusions:**

SEYLE achieved its objective of recruiting a large representative sample of adolescents within participating European countries. Analysis of SEYLE data will shed light on the effectiveness of important interventions aimed at improving adolescent mental health and well-being, reducing risk-taking and self-destructive behaviour and preventing suicidality.

**Trial registration:**

US National Institute of Health (NIH) clinical trial registry (NCT00906620) and the German Clinical Trials Register (DRKS00000214).

## Background

In the transition from childhood to adulthood adolescents make lifestyle choices and initiate patterns of behaviour that affect both their current and future well-being and health [1-6]. Many adverse health behaviours emerge in adolescence and track into adulthood, with increasing consequences for negative and sometimes long-lasting outcomes. Given the importance of this transitional period, it is essential to systematically assess the mental health and well-being of adolescents and young adults, and to implement and evaluate interventions for at-risk individuals. Several large studies have been carried out, mostly in the US, to gather information on both healthy and risk behaviours as well as psychiatric symptoms, based on robust methodologies [7-11]. Other studies analysed the effects of interventions to promote mental health and prevent suicide among adolescents [12-14].

However, to the best of our knowledge, no previous study compared the effectiveness of interventions based on different approaches with a Randomized Controlled Trial (RCT). The Saving and Empowering Young Lives in Europe (SEYLE) project was designed with this in mind.

SEYLE, supported by the European Union Seventh Framework Program (FP7), (Grant agreement number HEALTH-F2-2009-22309), is an RCT to evaluate school-based preventive interventions of risk-taking and self-destructive behaviours in eleven European countries, including: Austria, Estonia, France, Germany, Hungary, Ireland, Israel,^a^ Italy, Romania, Slovenia and Spain, with the National Centre for Suicide Research and Prevention of Mental Ill-Health (NASP) at Karolinska Institutet (KI) in Sweden responsible for the scientific coordination of the project. The Child Psychiatric Epidemiology Group at Columbia University and New York State Psychiatric Institute served as methodological experts. SEYLE is registered in both the US National Institute of Health (NIH) clinical trial registry (NCT00906620) and the German Clinical Trials Register (DRKS00000214). The full protocol of the study has been previously published [15]. The key objectives of the study were: (i) to collect assessment data on a cohort of European adolescents, including demographic information, psychopathology, lifestyles, values and risk-behaviours, in order to produce an epidemiological database on the general health status of European adolescents; (ii) to evaluate three types of school-based interventions in comparison to a minimal intervention control group. The three active interventions included (1) teacher training, (2) increasing adolescents’ awareness about mental health, and (3) professional screening of adolescents for mental health problems and risk behaviours. Teachers were trained through the gatekeeper program: Question, Persuade and Refer, developed in the US by the QPR Institute [16]. Pupils were trained through a standardized awareness-increasing program [15,17] designed to promote knowledge of mental health, healthy lifestyles and behaviours among adolescents. A professional screening program performed by psychiatrists and psychologists was specifically designed for the SEYLE study. All pupils were screened with a questionnaire and, if responses exceeded a predetermined cut-off score for depression, anxiety, phobia, alcoholism, substance abuse, non-suicidal self-injury (NSSI) or suicidality, pupils were interviewed and then referred for professional treatment if necessary. More details about the SEYLE interventions have been previously published [15].

The objectives of this article are to describe: 1) the study sites; 2) the main methodological issues employed; 3) the characteristics of the recruited sample including its representativeness; and 4) the internal reliability of the psychometric scales utilized for evaluation of the outcomes of the RCT: the Zung Self-Rating Anxiety Scale (Z-SAS, [18]), the Beck Depression Inventory, Second Edition (BDI-II, [19]), the World Health Organization Well-Being Scale (WHO-5 [20], and the Strengths and Difficulties Questionnaire (SDQ, [21].

## Methods

### Study sites

SEYLE had one study site in each of the eleven European countries described above. At each site at least one study catchment area, reasonably consistent with an administratively established geographic area was selected. The selected catchment areas in each country are described in Table 1. To meaningfully interpret the potential representativeness, key parameters, such as mean age, number of immigrants, population density, net income and gender proportion for each site were compared to the corresponding national data. Data at the national and local levels were extracted from Eurostat [22] and collected for each participant site. Effect sizes of mean age and number of immigrants at the country and study site levels were calculated for each country according to Cohen’s d, measured as small (d=0.3), medium (d=0.5) and large (d=0.8). Differences in gender distribution among 15-year olds at the country and study site levels were evaluated with a test of proportions. Population density and net income were compared between each country’s national data and the respective study site.

**Table 1:** **Demographics of SEYLE study sites, according to Eurostat**^**1-2**^

**Country**	**Study site**	**Population**	**Mean age**^**3**^	**% females**	**Pop. density**	**Income (net,EURO per year) **^**4**^	**Eurostat area**
Austria	Tirol	704,472	39.9	51.1	56.1	22,192	Tirol
Estonia	Tallinn	524,938	-	54.0	122	7,905	Põhja-Eesti
France	Lorraine	2,348,384	40.3	51.2	99.7	19,182	Lorraine
Germany	Heidelberg	10,749,506	41.8	50.8	300.7	24,719	Baden-Württemberg
Hungary	Budapest	2,925,500	40.7	53.3	179.4	8,735	Közép-Magyarország
Ireland	Cork and Kerry	648,700	-	50.7	53.5	-	Ireland South West
Italy	Region Molise	320,795	44.0	51.4	73.4	14,315	Molise
Romania	Cluj and Maramures counties	2,721,468	38.7	51.3	77.8	2,755	Romania Nord-Vest
Slovenia	Osrednjeslovenska, Podravska and Obalno-kraška region	965,200	-	50.8	155.4	9,889	Podravska, Osrednjeslovenska and Obalno-kraska
Spain	Oviedo, Gijon and Aviles	1,058,923	45.3	52.2	101.9	14,767	Principado de Asturias

^1^Eurostat. Statistics database. European Union; 2010. Available from: http://epp.eurostat.ec.europa.eu/portal/page/portal/eurostat/home/.

^2^Data from Israel is not included as the study site was the whole country.

^3^Not available for study sites in Estonia, Ireland and Slovenia.

^4^Not available for study site in Ireland.

### School and participant selection

At each site, eligible schools were randomly selected to participate in SEYLE. A list containing all available schools was generated at each site, and the schools were categorized as large or small and randomized into one of the four study arms for possible inclusion according to a randomized order. Simple randomization was used as a method of randomization of schools through a random number generator. Schools were categorized as small if they had less than or equal to the median number of students in all schools in the study area/region; and large if they had greater than the median number of students in all schools in the study area/region. Schools were considered eligible if they were public, contained at least forty 15-year-old pupils, had more than two teachers for pupils 15 years of age and no more than 60% of the pupils were of the same gender. These inclusion criteria were selected to allow for the recruitment of a comparable sample of schools and pupils across study sites, in spite of differences in sociocultural factors and in the organization of the educational system. However, a few exceptions were made in the case of sociocultural particularities of a specific country’s education system and applying the exact same criteria would increase selection bias instead of reducing it across sites. In particular in Ireland, single gender schools were allowed to participate in pairs with a single gender school of the opposite sex and of similar size. In Germany, due to the unique design of the school system, a sample of schools in the three categories of German high schools were selected and randomized separately. National and/or regional school authorities were contacted and informed about the project in general terms in order to get approval, which was obtained in all participating countries. The representatives of SEYLE at each study site then met with the school principals in the respective areas to describe the intervention of the Arm to which their school had been randomized and to explain the general objectives and procedures of that Arm. Each school was selected to participate in one Arm only and no information was disclosed about the interventions to be performed in other Arms of the RCT. On the basis of general information about SEYLE objectives and specific information about the specific intervention Arm into which the school was randomized, the school could accept or refuse to join. When a school refused to participate, the next school randomized in the same category was approached to replace it. It is important to note that schools were replaced only with other schools that were already in the randomization list. This procedure was designed to generate a balanced number of large and small schools in each intervention Arm, to minimize bias and increase the validity of the results. Within each school, all classes with a majority of 15 year olds were approached for participant recruitment, with a minimum of two schools per Arm. This procedure was repeated until a minimum of 250 students were recruited in a each Arm. Prior to requesting consent from the parents and assent from the pupils, general information about the SEYLE study and details about the specific Arm they were invited to participate in, was provided. Not all pupils for whom parental consent and adolescent assent were obtained actually participated, as some students were absent from school on the day the questionnaire was administered. Consent rates were calculated as the percentage of approached pupils for whom parental consent and pupil’s assent were both given. Participation rates were calculated as the percentage of assented pupils with parental consent who actually took part in the baseline questionnaire. In order to evaluate the impact of the consent rates of schools and pupils on the external validity of the collected data; school size, in terms of number of attending pupils, was compared between participating and non-participating schools. Moreover, gender proportion of pupils with and without consent were compared. Drop-out rates were calculated as the number of pupils assessed at baseline who did not participate at the first (3-months) and/or second (12-months) follow-up. Sociodemographic variables obtained at baseline and average scores on the scales employed, were used to evaluate differences between Arms.

### Instruments and interventions

A full description of assessment instruments and interventions was previously published [15].

### Standardization of methodology

Each SEYLE site used the same methodology in an effort to obtain comparable study results. Homogenous methodology was achieved through two different means. First, a detailed procedures manual (328 pages) was developed, containing information regarding every aspect of the study implementation, including school selection, recruitment, randomization, clinical backup, ethical issues, translation procedures and methods of cultural adaptation, detailed descriptions of each intervention and intervention time-lines, as well as the baseline and follow-up questionnaires. Second, uniform training procedures were conducted. All site leaders were initially trained centrally, in Stockholm. Site leaders then conducted local training for their own teams that included a minimum of 27 hours of group work, with at least 4 hours devoted to each intervention. To ensure study fidelity to the methodology, a series of monitoring site visits were carried out by representatives of the coordinating centre (NASP), together with the two consultants from Columbia University visiting each site. The site visits took place to overlap with training by site leaders and consisted of two-day consultations with local staff involved in the SEYLE project. Present at the site visit were the Intervention Arm coordinators, as well as the site leader (Table 2). During the site visit, local staff were required to present their understanding of the study and its procedures, as described in the manual, as well as the requirements of conducting each of the interventions. Site visits also provided an opportunity to correct any misunderstandings and to provide additional training, if necessary, to assure adherence to the protocol.

**Table 2:** SEYLE study key personnel

**Executive committee**							
Coordinator and Project Leader	Danuta Wasserman	National Centre for Suicide Research and Prevention of Mental Ill-Health (NASP) at Karolinska Institutet (KI), Stockholm, Sweden					
Deputy Coordinator	Marco Sarchiapone	Department of Health Sciences, University of Molise, Campobasso, Italy					
Project Manager and Assistant Project Leader	Vladimir Carli	National Centre for Suicide Research and Prevention of Mental Ill-Health (NASP) at Karolinska Institutet (KI), Stockholm, Sweden					
Consultants for Methodology	Christina Hoven	Department of Child and Adolescent Psychiatry, Columbia University-New York State Psychiatric Institute, New York, US					
	Camilla Wasserman						
**Intervention arm coordinators**							
QPR	Vladimir Carli	National Centre for Suicide Research and Prevention of Mental Ill-Health (NASP) at Karolinska Institutet (KI), Stockholm, Sweden					
ProfScreen	Romuald Brunner / Michael Kaess	Clinic of Child and Adolescent Psychiatry, Centre of Psychosocial Medicine, University of Heidelberg, Heidelberg, Germany					
Awareness	Camilla Wasserman	Department of Child and Adolescent Psychiatry, Columbia University-New York State Psychiatric Institute, New York, US					
Minimal Intervention/ Control	Marco Sarchiapone	Department of Health Sciences, University of Molise, Campobasso, Italy					
**Study sites**							
**Country**	**Site leader**	**Site coordinator**	**Arm coordinators**			**Translation coordinator**	**Workpackage leadership**
			***QPR***	***ProfScreen***	***Awareness***		
Austria	C. Haring	P. Olesky	C. Pajek	P. Olesky	C. Haring	-	-
Estonia	A. Värnik	R. Soonets	M. Sisask	L. Heidmets	R. Soonets	K. Valling	P.Varnik (Data management)
France	JP. Kahn	F. Guillemin	A. Tubiana	H. Vann	B. Bucki	JP Kahn	-
Germany	R. Brunner	M. Kaess	N. Schönbach	M. Kaess	K. Klug	M. Kaess	R. Brunner (Intervention Coordinator)
Hungary	J. Balazs	J. Balazs	M. Balint	G. Meszaros	L. Farkas	J. Balazs	J. Balazs (Translation procedures)
Ireland	P. Corcoran	H. Keeley	C. McAuliffe	F. Elahi/	J. McCarthy	H. Keeley	L-A. Burke (Analysis of cost-effectiveness)
				P. Cotter			
Israel	A. Apter	D. Feldman	C. Burzstein	S. Hen-Gal	Y. Apter	Y. Apter	D. Feldman (Quality control)
Italy	M. Sarchiapone	G. Nicolais	V. Carli	F. Basilico	M. Iosue	M. Sarchiapone	M. Iosue (Dissemination)
Romania	D. Cozman	B. Nemes	O. Dobrescu	B. Nemes	D. Herta	B. Nemes	D. Cozman (Materials for Schools)
Slovenia	V. Postuvan	V. Postuvan	U. Mars	T. Podlogar/	V. Postuvan/	V. Postuvan	-
				V. Košir	J. Žiberna		
Spain	J. Bobes	P. Saiz	E. Diaz-Mesa	M. Garrido	S. Al-Halabi	P. Saiz	P. Saiz (Cultural adaptation)
Sweden	D. Wasserman	V. Carli	-	-	-	-	D. Wasserman (Project coordination and data analysis)
**Administrative assistants**							
Tony Durkee	National Center for Suicide Research and Prevention of Mental Ill-Health (NASP) at Karolinska Institutet (KI)						
Brigit Frisen-Andersson							
Pierre Bodin							
Anna Lundgren							
External Ethical Advisor	University Hospital Basel, Psychiatric Clinics of the University of Basel, IBMB, University of Basel, Switzerland						
Stella Reither-Theil							

Cross-site collaboration was an important study objective and was facilitated prior to data collection, throughout implementation and up until study completion. For example, most sites assumed primary responsibility for one major study requirement, called a Work Package (e.g., translations, cultural adaptation, quality control, ethical requirements, data management, etc.) and collaborated with each of the other sites concerning this specific topic. Some of the major collaborative efforts are described below.

### Quality control procedures

As part of the SEYLE project, a method for quality control was developed and implemented. A series of questionnaires were sent to intervention coordinators in each country in order to ensure that all preparatory procedures were correctly conducted and that the interventions implemented at each site were faithful to the initial intervention models of SEYLE.

Analysis of these data allowed for assessment of the degree of discrepancy between different sites and between implementation in each site compared with the SEYLE model, as well as the effect variations had on the projects overall results and conclusions. Three quality control assessment tools were used - I. A questionnaire administered during site visits to assess the preparedness for intervention implementation; II. A pre-intervention questionnaire focused on questionnaire coding and on specific requirements to be carried out prior to and immediately after the intervention, and; III. A post-intervention questionnaire focused on the implementation of each intervention. Analyses of these questionnaires showed very small differences between the sites in the implementation process and did not identify any major modification in the implementation of the interventions in any site.

### Translation and cultural adaptation

The Hungarian site coordinated the translation processes in collaboration with the site-specific translation coordinators and the coordinating centre in Sweden (NASP). Translation coordinators oversaw site-specific translations, back translations and pilot interviews of all SEYLE materials. All materials were forward and back translated in each participating language. German was used in both Germany and Austria but was translated by the German site. All SEYLE materials (instruments & intervention Arm packages) were developed originally in English and then translated into the following languages: Gaelic (Irish), German, Estonian, French, Hebrew, Hungarian, Italian, Romanian, Slovenian and Spanish. In order to confirm the quality of the translations, staff from each site reviewed all back translations, evaluated reports on the respective pilot interviews and provided feedback to the Hungarian site. The site translation coordinators also implemented cultural adaptation: primarily concerning local linguistic phenomena and expressions. Focus groups were then conducted at each site to provide feedback on the cultural adaptation resulting from the pilot testing. In the case of ambiguity, consultation with a cultural linguistic advisor was sought. Based on these procedures, culturally adjusted language replaced the original in the final versions. A report concerning language issues, including possible ambiguity was sent to the coordinating centre for resolution, when necessary.

The scales used in the SEYLE questionnaires were included in the officially translated and validated version, when available, in the respective language, e.g., the SDQ [21], the WHO-5 etc. [15]. If the scale was not available in the required language it was translated (and back-translated) for SEYLE, using the same procedure as for the other study materials. Internal reliability for all scales used in SEYLE was assessed through Cronbach’s alpha [23].

### Data entry and data quality control

Data were collected on paper questionnaires, with the exception of the Austrian site, where data were collected electronically for direct data entry. In Germany, data collection forms were scanned for automatic data entry while in the other nine countries manual independent double data entry procedures were followed. In those countries information was entered twice using the Statistical Software Package SPSS 17.0. The Estonian site, which was responsible for the data management procedures, provided continuous oversight to other centers and promptly responded to any queries arising during the data entry process. A two-stage data cleaning and quality control procedure was performed to guarantee clean and reliable data. The first stage of quality control was performed locally, based on the two data files generated through double data entry. These files were compared and inconsistencies were resolved by checking the paper material. Based on this corrective action an accurate data file was generated. The second stage of quality control was performed centrally at the Estonian site, by double-checking each local dataset, attempting to detect other errors, such as incompleteness (missing values), inconsistencies (incorrectly followed skip-outs), irregularities (numbers inserted in text variables), and out of range data (i.e. very large number of siblings or sexual partners). The results of these control procedures generated a list of queries that was sent for resolution to the specific site. After finalizing quality control procedures, the Estonian site pooled the data into one database for all respondents for each wave (i.e., baseline, 3-month and 12-month follow-up). Pooled databases for each wave were then merged into one longitudinal pooled database.

### Ethics and emergency issues

Ethical issues were discussed with an independent ethical advisor from Basel University in Switzerland. Each site obtained permission from the local ethics committee to implement the SEYLE study in their respective country. According to guidelines from the local ethics committees, after thorough examination of the SEYLE study objectives and procedures, decisions were made locally to obtain consent through an opt-in method (parents had to sign a consent form if they allowed their child to participate) or an opt-out method (parents had to sign a refusal form if they did not want their child to participate). Study subjects were then recruited into the study accordingly, after obtaining the required informed consents from parents and assent from pupils [24].

A specific procedure to identify and immediately assist emergency cases with acute suicidality was implemented at each site in all four Arms. A minimum set of requirements regarding the identification of emergency cases was followed by each center. However each centre had the opportunity to reinforce the ethical requirements, according to the indications of the local Ethics Committee. Emergency cases were identified through responses to two specific questions in the questionnaire: those who reported moderate or severe suicidal ideation in the previous two weeks, or those who reported attempting suicide in the previous two weeks. Subjects identified as emergency cases were followed-up by local SEYLE personnel until successful referral to the local healthcare system. However, since it was not possible to follow up individuals while in treatment due to confidentiality, it is not known if the clinical intervention had impact on the collected data. However, all emergency cases were allowed to participate in the active interventions and in the control arm. Therefore, these pupils are included in the total data set.

Subsequent to conducting SEYLE, an interdisciplinary workshop^b^ was held in the Psychiatric Clinics of the University Basel, to analyse ethical issues, especially confidentiality towards minors involved in SEYLE Study. Additionally, relevant codes and guidelines for guidance in mental health research with minors were analysed. While unresolved questions may remain, such as whether and when confidentiality might or should be overridden in cases of emergency [25], within the SEYLE study, all problems of confidentiality were determined to have been handled according to the indications of the local ethical committees and the local laws and regulations of the participating countries.

## Results

### Consent, participation and drop-out rates

Response rates for SEYLE are reported in terms of consent and participation rates for schools and pupils.

#### Schools

If a school refused to participate, no pupil in that school was approached. The school randomization with replacement methodology, however, required refusal schools to be replaced by the next school of the same size on the school randomization list. Response rates for SEYLE schools, by country, are reported in Table 3. A total of 264 schools were approached for participation. Of these, 179 schools accepted, with an overall response rate of 67.8%. However, the school response rate was 72.0% when Israel, the only study site to have a low response rate (37.5%), was excluded. School size, measured by the total number of students in the school, of participating and non-participating schools did not differ in any country, with the exception of Slovenia, where participating schools were smaller than non-participating schools.

**Table 3:** SEYLE study school response rates, including number randomized, approached and participated, by country

**Country**	**Randomized schools**	**Approached schools**	**Accepted to participate**	Response rate*
Austria	22	22	15	68.2%
Estonia	23	23	19	82.6%
France	25	25	20	80.0%
Germany	100	41	26	63.4%
Hungary	23	19	15	78.9%
Ireland	24	24	17	70.8%
Israel	32	32	12	37.5%
Italy	18	18	14	77.8%
Romania	27	19	16	84.2%
Slovenia	32	20	13	65.0%
Spain	23	21	12	57.1%
**Total**	**349**	**264**	**179**	**67.8%**

* percentage of approached schools that accepted to participate in the SEYLE study.

#### Pupils

Rates of pupils’ consent have been calculated for the eight countries (Estonia, Germany, Hungary, Ireland, Israel, Italy, Romania and Spain) that used similar ethical procedures in collecting pupils consent. The overall rate of consent in these eight countries was 76% (10,665 pupils with consent out of 14,086 approached). In the other three countries (Austria, France and Slovenia), extended procedures for collection of the informed consent were imposed by the local ethics committees (i.e., multiple forms to be signed; pupil could be enrolled only if both parents signed the form, etc.). This resulted in a consent rate of 23% (3,452 pupils with consent out of 14,803 approached) in these three countries. When combining these three countries with the other eight the overall rate of consent decreased to 49% (14,117 pupils with consent out of 28,889 approached). Of the total 14,117 pupils whose parents gave consent, 12,395 participated in SEYLE, yielding a participation rate of 87.8%. Gender proportion of consented and non-consented pupils did not significantly differ in any country with the exception of France and Slovenia, where more girls were present among participating pupils. Information regarding gender proportion of non-participating pupils was not available in Ireland and Germany.

Overall, in the 3 months follow-up assessment, 10,823 pupils participated and 9,846 pupils participated at 12 months. The overall 12-month drop-out rate from baseline was 20.6%, including a 12.7% at 3 months. The drop-out rate did not differ significantly between countries and ranged between a minimum of 18.6% in the Control Arm and a maximum of 23.2% in the Awareness Arm. The differences in the socio-demographic and psychopathological characteristics at baseline, between those who participated in all waves of data collection and those who dropped out, did not differ significantly between Arms (Table 4). All schools remained actively involved through the three waves of data collections with no school drop-out. Differences in the socio-demographic and psychopathological characteristics between those who participated in all waves of data collection and those who did drop out did not differ significantly between Arms.

**Table 4:** Participation in SEYLE according to Intervention Arm, including baseline, 3 and 12 month follow-up and drop out rates, by gender

**Intervention arm**	**Gender**	**Baseline number (gender %)**	**3 Month follow-up number (gender %)**	**3 Month drop-out rate^ (%)**	**12 Month follow-up n (gender %)**	12 Month drop-out rate* (%)
QPR	Males	1323 (43.6)	1158 (43.1)	12.5	1043 (43.3)	21.2
	Females	1694 (55.8)	1515 (56.3)	10.6	1352 (56.1)	20.2
	Both genders	3036	2689	11.4	2410	20.6
Awareness	Males	1351 (44.6)	1106 (43.4)	18.1	979 (42.1)	27.5
	Females	1664 (54.9)	1430 (56.1)	14.1	1333 (57.2)	19.9
	Both genders	3032	2551	15.9	2329	23.2
ProfScreen	Males	1301 (42.4)	1158 (42.1)	11.0	1024 (41.7)	21.3
	Females	1752 (57.1)	1583 (57.5)	9.7	1423 (58.0)	18.8
	Both genders	3070	2752	10.4	2455	20.0
Minimal Intervention	Males	1554 (47.7)	1323 (46.7)	14.9	1239 (46.7)	20.3
	Females	1689 (51.9)	1494 (52.8)	11.6	1403 (52.9)	16.9
	Both genders	3257	2831	13.1	2652	18.6
Total		12395	10823	12.7	9846	20.6

*From baseline.

### Sample characteristics

#### Age and gender

The age and gender distribution of the sample, stratified by country, is shown in Table 5. Gender distribution of the 12,395 participating pupils was 6,799 females and 5,529 males (67 with missing gender data); the mean age was 14.91±0.90 (83 with missing age data). The largest sample was recruited in Germany (n=1444). Austria (n=960) was the only country that did not reach the target of 1000 pupils at baseline. Eight study sites recruited more females than males (Table 5). A larger number of males were recruited only in Ireland (54.7%), Israel (81.4%) and Spain (51.7%). In an analysis of representativeness, on the basis of Eurostat data [22], very small effect sizes were found concerning variations in the mean age between study sites and the respective country. Cohen’s d effect size also remained lower than 0.3 for the total sample when stratifying the analysis by gender. The largest effect size for both genders was found in Spain (d=0.205). For all other countries, the effect size of age was below 0.1. Differences in the proportion of 15-year old males and females and the respective country’s data were not statistically significant at any site. Analysis of representativeness was not conducted in Israel as the study site was of the entire country.

**Table 5:** SEYLE pupil participation by Country, according to mean age and gender

**Country**	**Pupils**		**Gender**			
	**N**^**a**^	**Mean age (SD)**	**Male**		**Female**	
			**N**^**b**^	**%**	**N**^**b**^	**%**
Austria	960	15.1 (0.8)	350	36.8	602	63.2
Estonia	1,036	14.2 (0.5)	477	46.0	560	54.0
France	1,000	15.2 (0.8)	319	31.7	688	68.3
Germany	1,444	14.7 (0.8)	692	47.9	752	52.1
Hungary	1,009	15.1 (0.8)	415	41.1	594	58.9
Ireland	1,091	13.7 (0.7)	600	54.7	496	45.3
Israel	1,256	15.9 (0.8)	1,023	81.4	233	18.6
Italy	1,189	15.3 (0.7)	381	32.0	811	68.0
Romania	1,139	15.0 (0.4)	395	34.6	745	65.4
Slovenia	1,165	15.2 (0.7)	347	29.7	823	70.3
Spain	1,023	14.5 (0.7)	530	51.7	496	48.3
Total	12,312	14.9 (0.9)	5,529	44.8	6,799	55.2

^a^83 pupils with missing age data have been excluded.

^b^67 pupils with missing gender data have been excluded.

#### Population density

Population density at the study sites was higher than in the respective country in Estonia, Germany, Hungary, Ireland and Spain. Population density was lower at the study site in Austria, France, Italy, Romania and Slovenia.

#### Income

The difference in net income per inhabitant between each country and the respective study site was below 10%, with the exception of Estonia (+17%), Germany (+15%), Hungary (+42%) and Italy (-24%).

#### Immigrants

The proportion of immigrants in each study site population was not significantly different from the proportion of immigrants in the respective country in all countries with the exception of Italy (-5%), Slovenia (-8%), and Spain (-10%).

#### Unemployment rates

In no country were unemployment rates at the study site significantly different than in the respective country as a whole.

Therefore, based on these key parameters, the pupils participating in the SEYLE study can be considered reasonably representative of their respective country.

Additionally, the main socio-demographic indicators such as age, gender, belonging to a single parent household, belonging to a religious denomination and parental unemployment did not significantly differ between Arms.

### Internal reliability of psychometric scales

The internal reliability of each scale was assessed separately for each country. The results are reported in Table 6. The internal reliability for the Z-SAS [18], the BDI-II [19], the WHO-5 [20] and the SDQ [21] was high or very high in most countries.

**Table 6:** Cronbach alpha of scales administered in the SEYLE study, by country (n=12,395)

**Country**	**Z-SAS**	**BDI-II**^**1**^	**WHO-5**	**SDQ**
Austria	.826^3^	.871	.752	.876
Estonia	.803^3^	.849^3^	.760^3^	.839^3^
France	.844^3^	.869	.810	.824
Germany	.829^3^	.875	.746	.789
Hungary	.811^3^	.835^3^	.796	.730
Ireland	.821	.872	.804	.848
Israel	.783^3^	.890^3^	.907^3^	.863^2^
Italy	.638^3^	.806	.765	.717
Romania	.811^3^	.864^3^	.748^3^	.806^3^
Slovenia	.855^3^	.867	.734	.716
Spain	.773^3^	.872	.773	.612
**Total**	**.805**	**.864**	**.799**	**.740**

*Z-SAS*, Zung Self-Rating Anxiety Scale; *BDI-II*, Beck Depression Inventory, Second Edition; *WHO-5*, World Health Organization Well-Being Scale; *SDQ*, Strengths and Difficulties Questionnaire;

^1^Item 21 of the BDI-II was not administered.

^2^Item 6 not administered in Israel and not included in the assessment of internal reliability.

^3^translated by the SEYLE study.

## Discussion

A large landmark intervention study, using RCT design, necessitates an article dedicated to the description of methodological issues and their complexity, which requires more space than is usually allowed in the Methods section of an ordinary article in the majority of scientific journals. This paper describes the complex methodological issues in the SEYLE study, which will allow for adequate interpretation of study’s results generated over-time, as well as appropriate replication and development of the study in the future.

SEYLE is a multi-site RCT of interventions to promote mental health and prevent risk behaviours and suicide in European schools. Very few RCTs have been conducted on youth mental health and most of them have focused on a single intervention or treatment method on a small sample or at only one site or within only one country, or alternatively with a clinical population [26-35]. SEYLE was designed to evaluate three different active intervention methods that respectively empower students, teachers and professionals, compared to controls, to identify early mental health problems and risk behaviours, while facilitating appropriate referral to the healthcare system. The interventions were performed on a large sample (N=12,395) in eleven sites, located in eleven different European countries. Extensive procedures were implemented in order to guarantee a homogeneous methodology across sites, including high quality forward and back-translations of manuals, instruments, standardized interventions, as well as cultural adaptation for each participating country and expert review of all ethical issues related to the investigation.

The SEYLE project achieved the sampling size objective of enrolling at least 1,000 participating pupils at each site (except Austria; n=960), for a total sample of N=12,395 school-based adolescents. Female participants (55.2%) exceeded the number of male participants. It may be hypothesized that girls are more interested and/or collaborative in participating in a study dealing with psychological issues than males, leading to a higher participation. However, in most countries, there were no significant differences between the gender proportion in the school and the gender proportion in our sample.

Analysis of representativeness indicates that the study sites are reasonably representative of their respective countries, thus allowing for in-country and between-country comparisons. The overall response rate of schools was high (67.8%). Only Israel had a low response rate of schools (37.5%). Without Israel, response rate of schools was 72%. It can be hypothesized that the low response rate of schools in Israel was attributed to the nearly uniform attitude of school principals’ against using school time for additional non-educational activities, in view of the many such activities already taking place. Israel, along with Cork, Ireland and Oviedo, Spain, were the only sites where a majority of the participating adolescents were male. The Cork study site had the lowest pupil participation rate (64.6%), which can possibly be attributed to factors outside the scope of the study, as an environmental emergency affecting the region (flooding) at the time of the SEYLE study, thus preventing many pupils attending school when the baseline questionnaire was administered. However, overall pupil participation rates in SEYLE were high and thus assure adequate external validity of the collected data.

Drop-out rates at follow-up were low: 20.6% at 12-months, including 12.7% at three months follow-up, indicating broad acceptance of the interventions and questionnaires by both schools and pupils. Drop-out rates did not vary significantly among countries. Importantly, the study methodology required that the school randomization include all eligible schools in the area. This allowed for comparability of study Arms within and across sites. The main demographic indicators at baseline, such as mean age, family structure and parental unemployment did not differ significantly between the Active interventions and the Control Arm.

Internal reliability of each scale administered in each country also provides reassuring results. Cronbach’s alpha values were measured for both instruments translated for the purposes of SEYLE, as well as for instruments already available officially, in the respective languages. As reported in Table 6, Cronbach’s alpha values were quite homogenous across countries with very small variations and can be considered good or very good for all administered scales. The lowest internal reliability was reported for the SDQ (alpha=0.740). This result is more than acceptable and in agreement with previous studies [36].

### Strengths

The major strength of the SEYLE RCT is its application of a robust and homogenous methodology applied across eleven study sites in eleven different countries, selected to provide a broad geographical representation of Europe. Due to extensive collaboration across sites through Work Packages, that required cross-site cooperation of all participating sites throughout the study, uniform adherence to the study methodology was assured. Moreover, the standardized translation methodology and cultural adaptation allowed for the fine-tuning of interventions to be responsive to local cultural contexts, thus ensuring that the project was meaningful and useful data were collected at each site. Another major strength of the project is the inclusion of a control group and the selection of outcome measures, which are related to mental health and wellness, as well as risk behaviours, thus allowing for the study outcomes to be associated with three distinct interventions. Finally, the SEYLE interventions are able to be tested on a combined, large sample of European adolescents, generating the first such findings from a large-scale RCT of adolescent well-being in Europe, providing an important cohort that can be followed over time.

### Limitations

In any large-scale multi-site study using a complex methodology, securing sufficient funding is always an important challenge. In the case of SEYLE, there were two major limitations due to funding: namely, the funding duration precluded a long follow-up after the intervention ended. It would have been of greater value to identify the long-term effects of the SEYLE interventions by having a longer follow-up, as many preventive effects may only be observed after a longer time post-intervention. In fact, a five-year instead of a three-year timetable for SEYLE would probably have allowed for more knowledge to be gained regarding the study’s outcomes. In SEYLE, one site per country was chosen for study participation. Sufficient funds to allow the inclusion of more than one site per country would significantly have improved representation of the urban and rural areas and therefore understanding of different populations. Moreover, the analysis of representativeness of the recruited sample in relation to the respective country was limited by the availability of sociodemographic indicators in Eurostat at the local level (NUTS2). It was not possible to directly compare the SEYLE data and the same indicators at the country level because these were not available for the adolescent population or were collected with different methodologies, ultimately being incompatible.

Consent rates of schools and pupils varied across countries. The consent rates of pupils were very good in eight countries and lower in the three countries where extended consent procedures were imposed by the local ethics committees. However, it has been reported that response rates between 30% and 70% are, at most, only weakly associated with bias [37]. Available indicators such as school size did not differ significantly between participating and non-participating schools with the exception of Slovenia, where more small schools participated in the study. The study was necessarily performed during school hours and consequently there was limited opportunity to collect other than questionnaire data regarding pupil’s behaviour. This school-based approach necessarily required a very limited number of outcome measures. Another limitation is that all data were collected through self-report questionnaires.

## Conclusions

The SEYLE RCT study was successful in recruiting a reasonably representative sample of over 12,000 European school-based adolescents. The study is unique in its’ robust and uniform methodology applied across eleven sites, including a large number of socio-demographic, lifestyle and mental health outcomes, allowing for evaluation of the effects of three Intervention Arms compared to a Control Arm. Several important indicators, such as response and participation rates, differences between Arms and reliability of scales show very good validity of the collected data and ensure that the selected outcome measures are reliable and useful for carrying out school-based identification of at-risk adolescents. The SEYLE database contains up-to-date information about lifestyles and mental health problems of European adolescents and will be of great benefit for mental health professionals, policy makers and other stakeholders throughout the European Union.

## Endnotes

^a^Israel belongs to the WHO European Region and is eligible to receive funding under the European VII Framework Programme.

^b^Workshop (When) Theory meets Practice – Ethical Issues in Research with Minors and other Vulnerable Groups, 14.2.2012 Research Ethics – Botnar Project.

## Competing interests

The authors declare that they have no competing interests.

## Authors’ contributions

VC wrote most of the manuscript, including critical revision of the manuscript, participated in the design of the project and supervised data analysis. CW wrote several sections of the manuscript, revised it and provided critical input to the study design and the methodology. DW, the project leader and scientific coordinator of the consortium, devised the study design and methodology, and critically revised all the phases of the writing of the manuscript. MS participated in the design of the study and critically revised the manuscript. CH participated in the design of the study, provided consultation for epidemiological issues, advised on research methodology and critically revised the manuscript. AP, JBa, JBo, RB, PC, DC, CH, JPK, VP, AV, MS and DW are the site leaders for the SEYLE project in their respective countries. SRT is the expert ethical advisor for the SEYLE project, providing consultation for the ongoing interventions. The other authors are the site and/or Arm coordinators for the SEYLE center in their respective countries. All the authors critically revised the manuscript before submission.

## Pre-publication history

The pre-publication history for this paper can be accessed here:

http://www.biomedcentral.com/1471-2458/13/479/prepub
